# miR-9875 functions in antiviral immunity by targeting PDCD6 in mud crab (*Scylla paramamosain*)

**DOI:** 10.1080/21505594.2020.1787078

**Published:** 2020-06-28

**Authors:** Yi Gong, Tongtong Kong, Xin Ren, Shanmeng Lin, Shengkang Li

**Affiliations:** a Guangdong Provincial Key Laboratory of Marine Biology, Shantou University, Shantou, China; b Institute of Marine Sciences, Shantou University, Shantou, China; c Southern Marine Science and Engineering Guangdong Laboratory, Guangzhou, China; d STU-UMT Joint Shellfish Research Laboratory, Shantou University, Shantou, China

**Keywords:** miR-9875, PDCD6, apoptosis, WSSV, *Scylla paramamosain*

## Abstract

Programmed cell death 6 (PDCD6) is a well-known apoptosis regulator that is involved in the immunity of mammals. However, the effects of miRNA-mediated regulation of PDCD6 expression on apoptosis and virus infection in organisms, especially in marine invertebrates, have not been extensively explored. In this study, PDCD6 of mud crab (*Scylla paramamosain*) (*Sp*-PDCD6) was characterized. The results showed that *Sp*-PDCD6 contains five EF-hands domains and could suppress virus infection *via* apoptosis promotion. It also presented that *Sp*-PDCD6 was directly targeted by miR-9875 *in vitro* and *in vivo*, miR-9875 served as a positive regulator during the virus invasion. The findings indicated that the miR-9875-PDCD6 pathway possessed fundamental effects on the immune response to virus infection in mud crab. Therefore, our research provided a novel insight into the roles of both miR-9875 and PDCD6 in the regulation of apoptosis and virus defense in mud crab.

## Introduction

The exploration of the virus-host interactions during the viral infection is essential for development mitigation strategies. Generally, the innate immunity, including humoral and cellular immune responses, plays a crucially important role in recognizing and protecting the invertebrates against harmful microbes [1]. Is has been known that apoptosis is a kind of cellular immune response essential in the host antiviral immunity. Apoptosis is a form of programmed cell death relating to chromatin condensation, cytoplasmic shrinkage, and plasma membrane blebbing [2]. Several studies have demonstrated that viruses could induce the infected cell undergoing apoptosis in both vertebrates and invertebrates [3,4], and the host cells could prevent virus infection and limit virus replication *via* apoptosis [5,6]. So far, the relationships between apoptosis and viral infection have been previously focused on organisms, but not in marine invertebrates, especially in mud crab.

Programmed cell death 6 (PDCD6), also called apoptosis-linked gene-2 (ALG-2), is a calcium-binding modulator protein that contains 5 EF-hand motifs [7,8]. PDCD6 is a well-known apoptotic mediator, which has been reported to be able to interact with the human death-associated protein kinase 1 (DAPk1) and promote apoptosis *via* caspase-3 dependent pathway [9]. Besides, follicle-stimulating hormone (FSH) inhibits ovarian cancer cell apoptosis through the down-regulation of PDCD6 [10]. In addition, the previous studies have demonstrated that PDCD6 was functionally redundant [11], which could also be severed as an important regulator during the tumorigenesis of lung cancer and epithelial ovarian cancer through stimulating cell migration or invasion [12,13]. Recently, PDCD6 has been regarded as a significant prognostic biomarker for advanced gastric cancer patients [14]. Studies showed that PDCD6 could inhibit angiogenesis through PI3 K/mTOR/p70S6 K pathway *via* interacting with VEGFR-2 [15]. Moreover, PDCD6 is also involved in endocytosis and signal transduction in mammals [16]. At present, although PDCD6 has been previously studied in model organisms, little is known in marine invertebrates.

With the discovery of non-coding small RNAs, many research suggested that these non-coding small RNAs may play an important role in the bio-functional regulation of diverse animals [17]. MicroRNAs (miRNAs) are endogenous small non-coding RNA molecules that can downregulate the expressions of specific target genes through binding to the 3ʹUTR of mRNA and result in the translation repression or direct mRNA degradation of target genes [18,19]. It has been found that miR-124 can affect cell proliferation, cell cycle, apoptosis, migration, and invasion *via* inhibiting the expression of PDCD6 in ovarian cancer cells [20]. Additionally, miR-20a promotes cell growth by targeting PDCD6 in cervical carcinoma cells [21]. Thus, PDCD6 was able to be regulated by miRNAs during the bio-functional regulation. However, the regulation of PDCD6 (by miRNAs) and its roles in marine invertebrates has not been intensively investigated. In an attempt to explore the roles of PDCD6 in antiviral immunoregulation and its involvement in the regulation of miRNAs, the mud crab miRNA targeting PDCD6 was characterized in this study. As an infection model we used white spot syndrome virus (WSSV). This large enveloped double-stranded DNA virus is a pathogen influencing many marine crustaceans including shrimp, crayfish and crabs [22] and causes huge economic losses in marine aquaculture. The results of this study revealed that miR-9875 could directly target PDCD6 and the miR-9875-PDCD6 pathway may regulate the apoptosis and virus infection in mud crab.

## Materials and methods

### Mud crab culture and WSSV challenge

Healthy male mud crabs, approximately 50 g each, were taken from a local crab farm (Niutianyang, Shantou, Guangdong, China), the culture conditions in the crab farm were 10‰ salinity and 25ºC. Thus, the crabs were acclimated under laboratory conditions (10‰ salinity, 25ºC) for a week before further processing. Then 200 μL of WSSV (1 × 10^6^ copies/mL) was injected into the base of the fourth leg of each crab according to our previous study [23]. At 24 and 48 h post-infection, hemolymph was collected from three randomly chosen crabs per group for further investigations.

### Gene cloning

Total RNA was extracted from hemocytes using Trizol (Invitrogen), followed by reverse transcribed with PrimeScript^TM^ II 1^st^ Strand cDNA Synthesis Kit (Takara, Japan). The open reading frame (ORF) of *Sp*-PDCD6 was amplified *via* PCR with specific primers (PDCD6-F1, 5ʹ-AATCACCAACTCACAAGATGG-3ʹ and PDCD6-R1, 5ʹ-CCATCTTGTGAGTTGGTGATT-3ʹ). Purified DNA fragments were cloned into the pMD® 19-T vector (TaKaRa, Japan) and sequenced by a commercial company (BGI, Shenzhen, China).

### RNA interference of PDCD6

Based on the sequence of *Sp*-PDCD6 (GenBank accession number MH558574.1), the siRNA specifically targeted the *Sp*-PDCD6 gene was designed, generating PDCD6-siRNA (5ʹ-ACAUACUUCCAUAAAGCUCCAUU-3ʹ) and its control PDCD6-siRNA-scrambled (5ʹ-UCAACAUACAUUAAGACUCCCUU-3ʹ). The siRNA was synthesized using the *In vitro* Transcription T7 Kit (TaKaRa, Dalian, China) according to the manufacture’ s instructions. Then, 50 μg of PDCD6-siRNA or PDCD6-siRNA-scrambled was injected into each mud crab, respectively. At different time post-siRNA injection, three mud crabs were randomly selected for each treatment and stored at −80°C for later use.

### Quantification of mRNA with quantitative real-time PCR

Total RNA was extracted from hemocytes, followed by first-strand cDNA synthesis using the PrimeScript™ RT Reagent Kit (TaKaRa, Japan). The primers PDCD6-F2 (5ʹ-ACACCATTCAACCCAGATAC-3ʹ) and PDCD6-R2 (5ʹ-ATACTTCCATAAAGCTCCAA-3ʹ) were used to quantify the PDCD6 mRNA, while the primers β-actin-F (5ʹ-GCGGCAGTGGTCATCTCCT-3ʹ) and β-actin-R (5ʹ-GCCCTTCCTCACGCTATCCT-3ʹ) were used to quantify the internal control (β-actin). Relative fold change of mRNA expression level of PDCD6 was determined using the 2^−∆∆Ct^ algorithm.

### Analysis of WSSV copies by quantitative real-time PCR

To detect WSSV copies in mud crab, the qPCR analysis was carried out using Premix Ex Taq (Probe qPCR) (Takara, Dalian, China). The qPCR was performed with WSSV-specific primers WSSV-RT1 (5ʹ-TTGGTTTCATGCCCGAGATT-3ʹ) and WSSV-RT2 (5ʹ-CCTTGGTCAGCCCCTTGA-3ʹ) and a TaqMan probe (5ʹ-FAM-TGCTGCCGTCTCCAA-TAMRA-3ʹ) according to a previous study [24]. The internal standard of qPCR was a DNA fragment of 1400 bp from the WSSV genome [25].

### Detection of apoptotic activity

In order to evaluate the apoptotic activity of mud crab, the caspase 3/7 activity of hemocytes was determined using the Caspase-Glo 3/7 assay (Promega, USA). Besides, the apoptosis rate was evaluated using FITC Annexin V Apoptosis Detection Kit I (BD Pharmingen^TM^, USA) according to manufacturer’s instructions.

### Target gene prediction of miRNA

Targetscan (http://www.targetscan.org), miRanda (http://www.microrna.org/) and RNAhybrid (https://bibiserv.cebitec.uni-bielefeld.de/rnahybrid/) were used to predict the miRNAs that target PDCD6 by a commercial company (BioMarker, Beijing, China). The overlapped miRNAs (predicted by the three algorithms) were the potential miRNAs.

### Plasmid constructions

The PDCD6 3ʹUTR was constructed into a pIZ/V5-His vector (Invitrogen, USA) with primers5ʹ-GCGTCTAGAACAGCACCAAAAATAATGCATG-3ʹ and 5ʹ-ATACCGCGGTCAGTATATCTGTACAACACGC-3ʹ. The mutated sequence (ATGTGGA) of the PDCD6 3ʹUTR sequence complementary to the miR-9875 seed sequence (CCTCTTC) was a control and generated by 5ʹ-TAAGCACAACCATGTGG ACCTGTTCTCTT-3ʹ and 5ʹ- CCTTAAGGGTTGTGATAGTGGAGG -3ʹ.All the recombinant plasmids were confirmed by sequencing in a commercial company.

### Cell culture, transfection, and fluorescence assays

The *Drosophila* Schneider 2 (S2) cells (Invitrogen) were cultured in Express Five serum-free medium (SFM) (Invitrogen) at 27ºC. The EGFP-PDCD6 or EGFP-ΔPDCD6 plasmid (100 ng/well) and the synthesized miR-9875 (miR-9875-scrambled) (50 nM/well) were co-transfected into S2 cells using the Cellfectin II Reagent (Invitrogen, USA). After 48 h of co-transfection, the EGFP fluorescence of S2 cells was measured by a Flex Station II microplate reader (Molecular Devices, USA) at 490/510 nm of excitation/emission (Ex/Em).

### *Fluorescence* in situ *hybridization*

The hemocytes of mud crab were seeded onto the polysine-coated coverslips, fixed with 4% polyformaldehyde for 15 min at room temperature. After that, the coverslips were dehydrated in 70% ethanol overnight at 4°C, followed by incubation with hybridization buffer [1× SSC (15 mM sodium citrate, 150 mM sodium chloride, pH 7.5), 10% (w/v) dextran sulfate, 25% (w/v) formamide, 1× Denhardt’s solution] containing 100 nM probe for 5 h at 37°C. The miR-9875 probe (5ʹ-FAM-CTCCTCCCTTCCTCTTCC-3ʹ) and PDCD6 probe (5ʹ-Cy3-AGAGTTGTCGTTGTCGAATGAG-3ʹ) were used. Then the slips were washed with PBS three times, and the hemocytes were stained with DAPI (4ʹ, 6-diamidino-2-phenylindole) (50 ng/mL) (Sigma, USA) for 5 min [26]. The images were captured using a CarlZeiss LSM710 system (Carl Zeiss, Germany).

### The silencing or overexpression of miR-9875 in mud crab

Anti-miR-9875 oligonucleotide (AMO-miR-9875) or miR-9875 mimic was injected at 30 μg/crab to knockdown or overexpress the miR-9875 in mud crab. The AMO-miR-9875 (5ʹ-CTCCTCCCTTCCT**C**TT**C**C-3ʹ) and miR-9875 mimic (5ʹ- GGAAGAGGAAGGG**A**GG**A**G-3ʹ) were modified with 2ʹ-O-methyl (OME) (bold letters) and phosphorothioate (the remaining nucleotides). The randomly scrambled sequences of miR-9875 mimic (5ʹ-AGGGGGGAAAGGAAGGGA-3ʹ) and AMO-miR-9875 (5ʹ-TCCCCTTCCTCCT**C**CT**T**C-3ʹ) were used as controls, respectively. All oligonucleotides were synthesized by Sangon Biotech (Shanghai, China). At different time points after the last injection, three mud crabs per treatment were collected for later use.

### Quantification of miR-9875 with quantitative real-time PCR

Total RNA was extracted using MagMAX^TM^ mirVana^TM^ Total RNA Isolation Kit (Thermo Fisher Scientific, USA), followed by first-strand cDNA synthesis *via* PrimeScript^TM^ II 1^st^ Strand cDNA Synthesis Kit (Takara, Japan) using (5ʹ-GTCGTATCCAGTGCAGGGTCCGAGGTCACTGGATACGACCTCCTCCC-3ʹ). The qPCR was carried out using the Premix Ex Taq (TaKaRa, Japan) to quantify the expression level of miR-9875. U6 was used as a control. The primers, including miR-9875-F (5ʹ-CGCCGGGAAGAGGAAGGG-3ʹ) and miR-9875-R (5ʹ-TGCAGGGTCCGAGGTCACTG-3ʹ), U6-F (5ʹ-CTCGCTTCGGCAGCACA-3ʹ), and U6-R (5ʹ-AACGCTTCACGAATTTGCGT-3ʹ), were used in this study.

### Statistical analysis

All data were subjected to one-way ANOVA analysis using Origin Pro8.0, with *P* < 0.01 considered statistically significant. All assays were biologically repeated for three times.

## Results

### *Bioinformatics analysis of* Sp-*PDCD6 cDNA*

**Figure 1.**
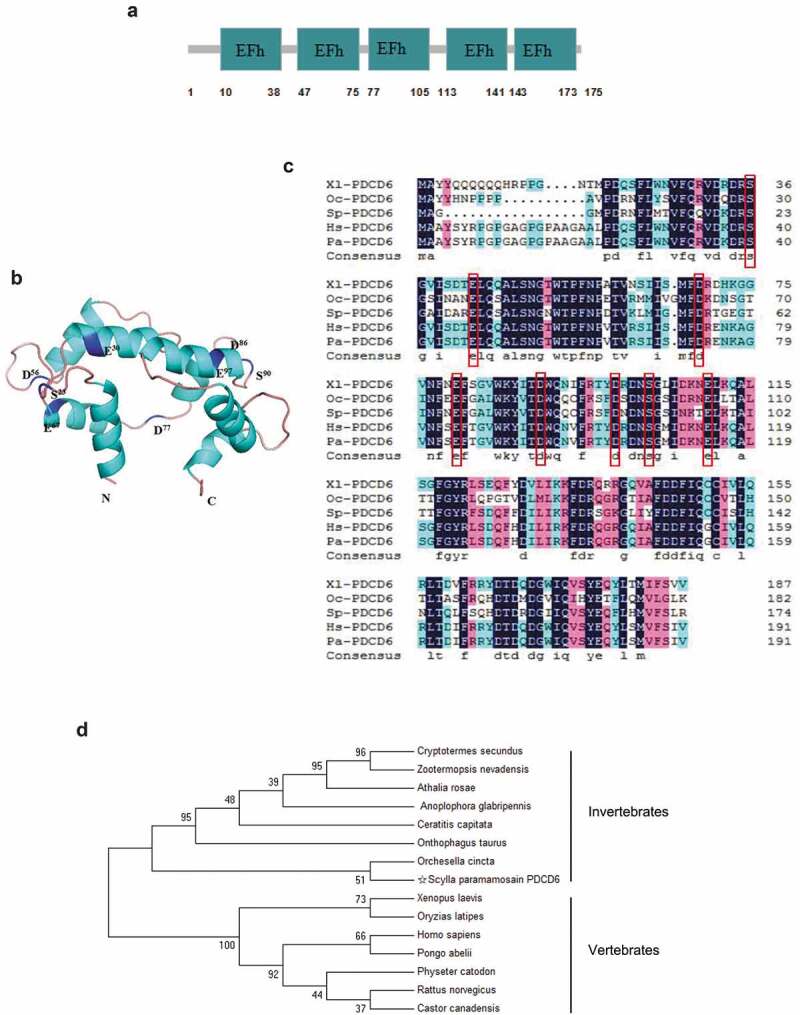
Bioinformatics analysis of PDCD6 (a) Schematic view of the structure of PDCD6 protein. EFh indicates EF-hands. (b) The three-dimensional model of PDCD6 protein. Eight conserved Ca^2+^-binding sites (S^23^, E^30^, D^56^, E^67^, D^77^, D^86^, S^90^, and E^97^) were marked in blue. (c) Protein sequence alignment of PDCD6 proteins with four other species from GenBank. Conserved Ca^2+^-binding sites were marked with the red box. Proteins analyzed listed below: Xl-PDCD6, *Xenopus laevis* PDCD6 (AAI10940.1); Oc-PDCD6, *Orchesella cincta* PDCD6 (ODM99366.1); Hs-PDCD6, *Homo sapiens* PDCD6 (NP_037364.1); Pa-PDCD6, *Pongo abelii* PDCD6 (XP_024102869.1). (d) Phylogenetic tree of aligned amino acid sequences of PDCD6. The black star marked *Sp-*PDCD6. The 1000 bootstraps were performed on the Maximum Likelihood phylogenetic tree to check the repeatability of the results.

The cDNA sequence of PDCD6 from mud crab (*Sp-*PDCD6) contains an ORF of 528 bp encoding 175 deduced amino acids. The putative *Sp-*PDCD6 protein possesses five EF-hands domains (Figure 1a) and eight conserved Ca^2+^-binding sites (S^23^, E^30^, D^56^, E^67^, D^77^, D^86^, S^90^, E^97^) (Figure 1b). Multiple alignments revealed a high homology among the amino acid sequences of *Sp-*PDCD6 from different species (Figure 1c). Phylogenetic tree analysis based on the amino acid sequences of *Sp-*PDCD6 and other species was constructed, and the data showed that *Sp-*PDCD6 had an evolutional relationship with the others and clustered together with the PDCD6 from invertebrates (Figure 1d). Besides, the cDNA sequence of *Sp-*PDCD6 has been deposited at GenBank under the accession number MH558574.1.

### Effects of PDCD6 on virus infection in mud crab

**Figure 2.**
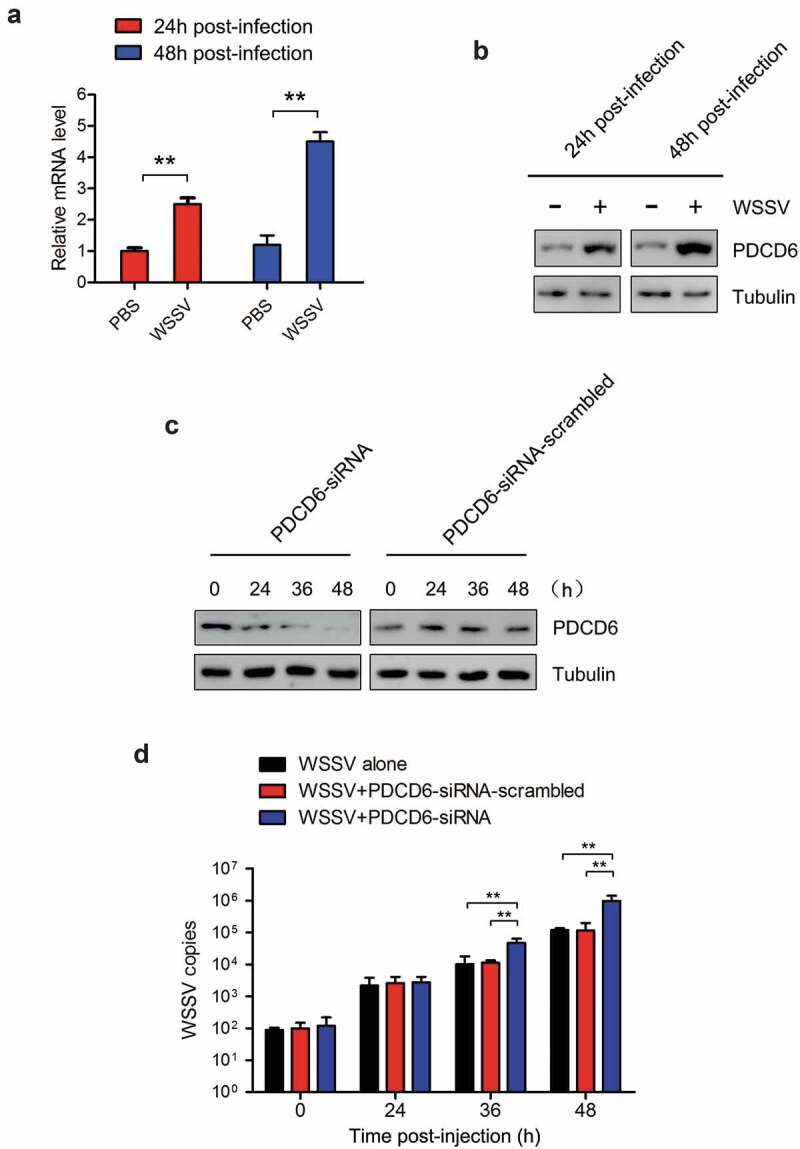
Effects of PDCD6 on virus infection in mud crab (a) Transcription levels of PDCD6 in WSSV-infected mud crabs. β-actin was used as an internal control. (b) Western blot analysis of PDCD6 expression in the hemocytes of WSSV challenged crabs. Tubulin was used as an internal control. (c) The efficiency of PDCD6 knockdown. At 24 h and 48 h post-PDCD6-siRNA treatment, the PDCD6 protein of hemocytes was detected by western blot. (d) The influence of PDCD6 silencing on WSSV infection in mud crab. WSSV and PDCD6-siRNA were co-injected into mud crab, followed by the detection of WSSV copy numbers. The values referred to the means ± standard deviation of triplicate assays (**, *p* < 0.01).

In order to determine the effect of PDCD6 on virus infection, mud crabs were challenged with WSSV and the expression of PDCD6 was detected. The results revealed that both mRNA and protein expression of PDCD6 was significantly elevated at 24 and 48 h post-WSSV challenge (Figure 2a and 2b), suggesting that PDCD6 might be involved in the immune responses to virus. To further ascertain whether PDCD6 could affect the proliferation of WSSV in mud crab, PDCD6-siRNA was injected into a mud crab to knockdown the expression of PDCD6. The knockdown efficiency of PDCD6 in the hemocytes of mud crab was determined by Western blot. The results showed that the protein level of PDCD6 in the PDCD6-siRNA injected group was significantly decreased compared with those in the PDCD6-siRNA-scrambled treated group (Figure 2c). WSSV and PDCD6-siRNA were co-injected into mud crabs, and the copy numbers of WSSV were detected. The results showed that the copy numbers of WSSV in the PDCD6 silenced mud crabs was significantly increased compared with the controls (Figure 2d). These data indicated that PCPD6 could suppress WSSV infection in mud crabs.

### The role of PDCD6 in regulating apoptosis of mud crabs

**Figure 3.**
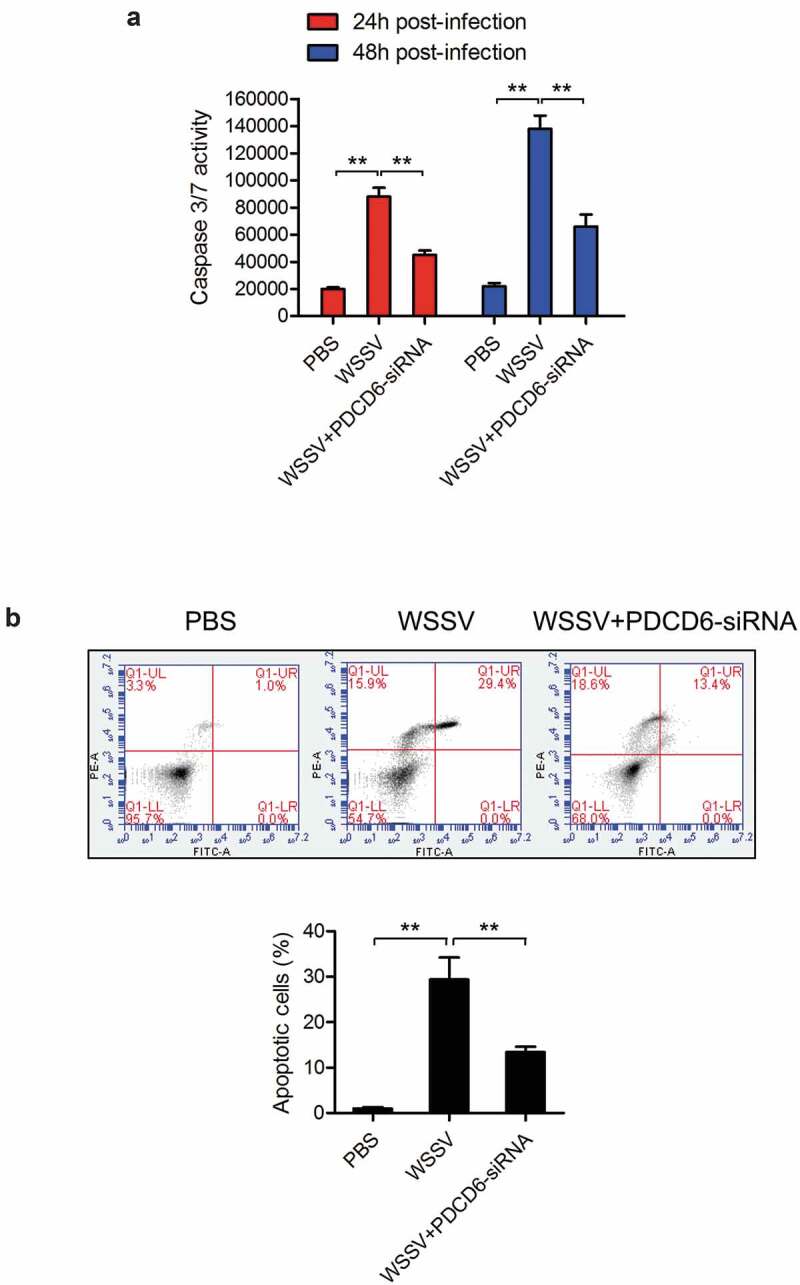
The role of PDCD6 in regulating apoptosis (a-b) The influence of PDCD6 on apoptosis of mud crab hemocytes. PBS or PDCD6-siRNA were co-injected with WSSV into mud crab, then, the apoptotic levels of the hemocytes were examined through the caspase 3/7 activity analysis (a) and annexin V assay (b). All data were the average from at least three independent experiments, mean ± s.d. (**, *p* < 0.01).

To explore the involvement of PDCD6 in the apoptosis during the PDCD6-mediated virus suppression, caspase 3/7 activity and apoptosis rate in the mud crabs treated with either WSSV or PDCD6-siRNA were determined. The results showed that the caspase 3/7 activity and apoptosis rate of mud crabs following WSSV challenge were significantly increased compared with the controls (Figure 3a and 3b), suggesting that WSSV could induce apoptosis in mud crabs. However, both caspase 3/7 activity and apoptosis rate in mud crabs treated with WSSV and PDCD6-siRNA were significantly decreased compared to such in mud crabs treated with WSSV only (Figure 3a and 3b). These results demonstrated that PDCD6 could suppress the virus infection through the induction of apoptosis.

### The interaction between miR-9875 and PDCD6

**Figure 4.**
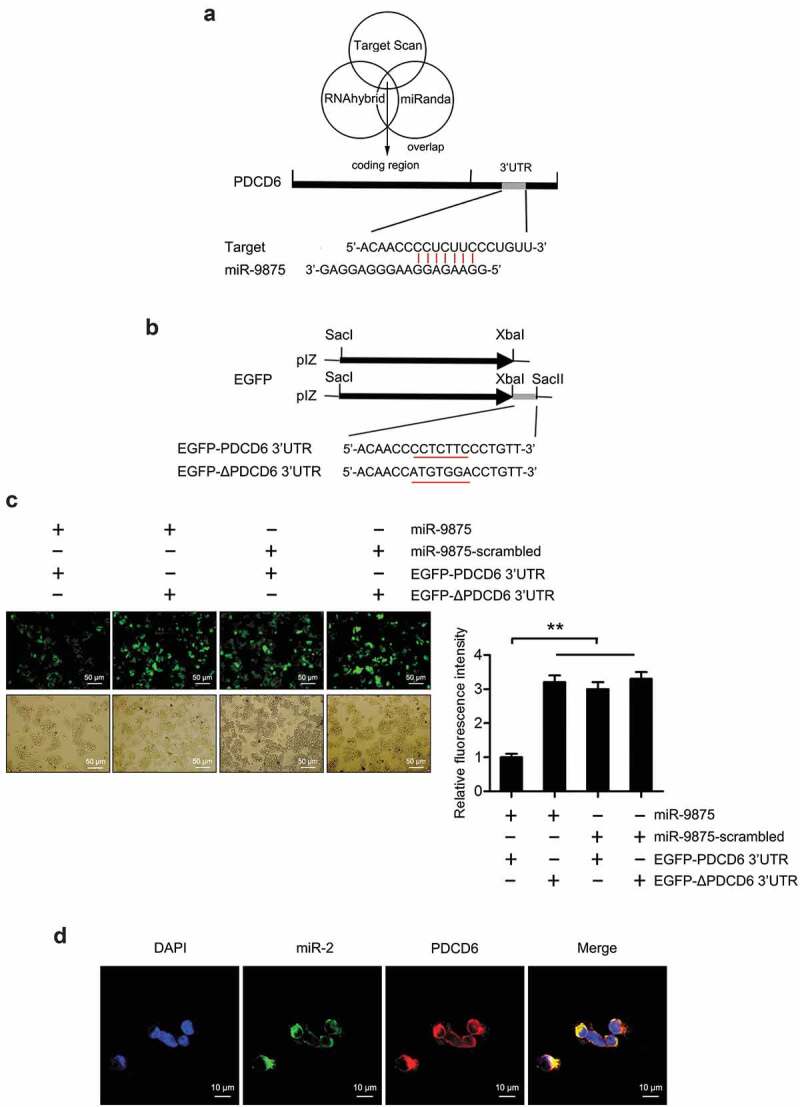
The interaction between miR-9875 and PDCD6 (a) The prediction of miRNA targeting PDCD6. Three algorithms were used for the prediction analysis, as predicted, miR-9875 could target the 3ʹUTR of PDCD6. (b) The construction of the plasmid EGFP-PDCD6 or EGFP-ΔPDCD6. The seed sequence targeted by miR-9875 was underlined. (c) The interaction between miR-9875 and PDCD6 in S2 cells. The indicated constructed plasmids (EGFP-PDCD6 and EGFP-ΔPDCD6) and miR-9875 or miR-9875-scrambled were co-transfected into S2 cells, then fluorescence intensity of cells was detected and analyzed. (d) The co-localization of miR-9875 and PDCD6 mRNA in the hemocytes of mud crabs. miR-9875, PDCD6 mRNA and nucleus of hemocytes were correspondingly detected with FAM-labeled miR-9875 probe (green), Cy3-labeled PDCD6 probe (red) and DAPI (blue). Scale bar, 10 μm. Each experiment was performed in triplicate and data are presented as mean ± s.d. (**, p < 0.01).

To reveal the role of miRNA in regulating the expression of PDCD6 gene, the miRNAs targeting PDCD6 were predicted. The results showed that miR-9875 could target PDCD6 in mud crabs (Figure 4a). To assess the interaction between miR-9875 and PDCD6, the plasmid EGFP-PDCD6 consisting of EGFP and PDCD6 3ʹUTR was constructed, and the plasmid EGFP-ΔPDCD6 was used as a control (Figure 4b). Then, the constructed plasmids and miR-9875 were co-transfected into S2 cells. The results indicated that the fluorescence intensity of the cells co-transfected with EGFP-PDCD6 and miR-9875 was significantly decreased compared with the controls, indicating that miR-9875 inhibited the expression of the PDCD6 gene by targeting its 3ʹUTR (Figure 4c). Furthermore, fluorescence *in situ* hybridization was used to detect the subcellular location of miR-9875 and PDCD6 in the hemocytes of mud crabs. The results revealed that miR-9875 was co-localized with PDCD6 mRNA in the hemocytes of mud crabs (Figure 4d). The above findings suggested that PDCD6 was the direct target gene of miR-9875.

**Figure 5.**
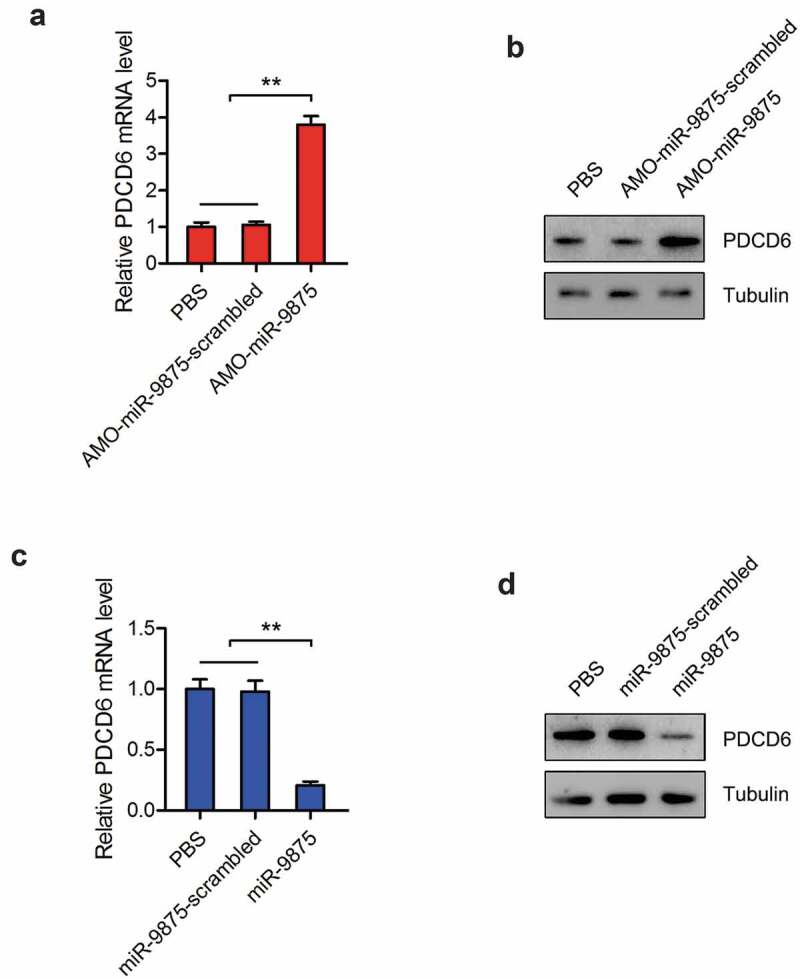
Impact of miR-9875 on PDCD6 expression in mud crab (a-b) The effects of miR-9875 silencing on the expression levels of PDCD6 in mud crabs injected with either AMO-miR-9875 or AMO-miR-9875-scrambled; the mRNA (a) and protein (b) expression levels were examined at 48 h post-injection. (c-d) The effects of miR-9875 overexpression on the expressions of PDCD6 in mud crabs injected with either miR-9875 or miR-9875-scrambled; the mRNA (c) and protein (d) expression levels were examined at 48 h post-injection. Significant statistical differences between treatment were indicated with asterisks (**, *p* < 0.01).

To explore the interactions between miR-9875 and PDCD6 *in vivo*, the expression of miR-9875 was silenced or overexpressed in mud crabs. The expressions of PDCD6 were determined and the results showed that the mRNA and protein expressions of PDCD6 were significantly increased in the miR-9875 knockdown mud crabs compared with the controls (Figure 5a and 5b). However, the mRNA and protein levels of PDCD6 was remarkably decreased when miR-9875 up-regulated (Figure 5c and 5d). These data demonstrated that miR-9875 interacted with PDCD6 in mud crabs.

### The influence of miR-9875-PDCD6 pathway on apoptosis regulation

**Figure 6.**
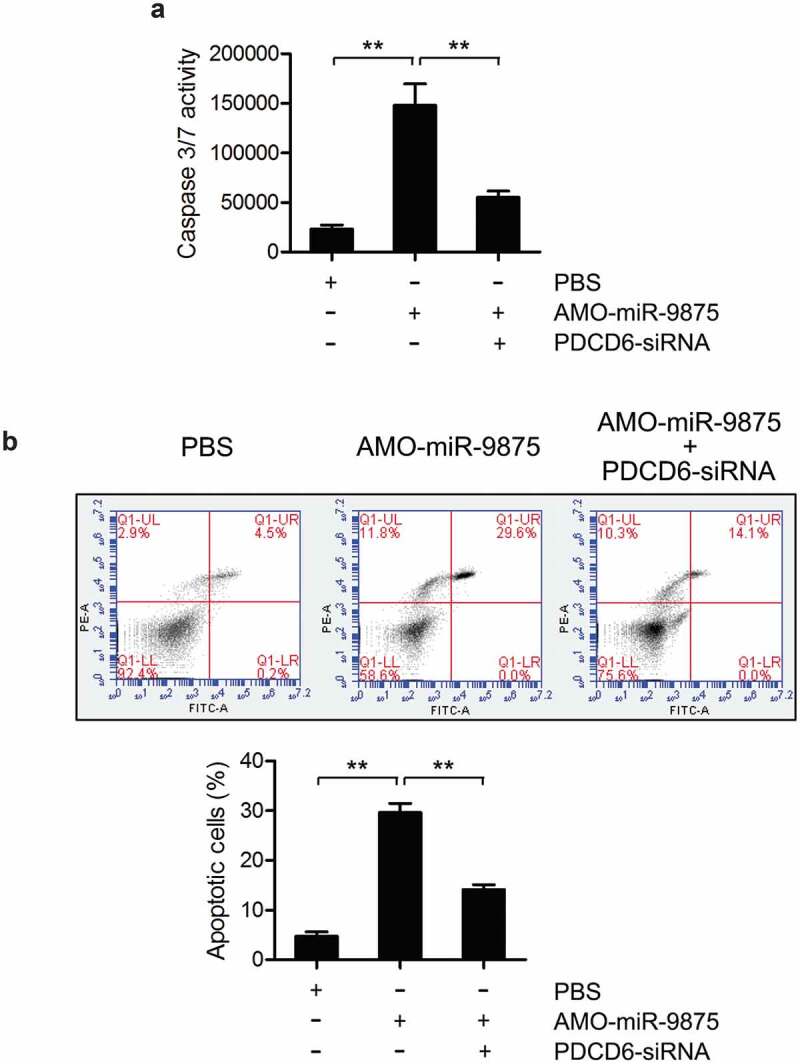
Suppression of apoptosis activity by miR-9875 *via* targeting PDCD6 (a) The caspase 3/7 activity in mud crab treated with either AMO-miR-9875 or co-treated with AMO-miR-9875 and PDCD6-siRNA. (b) The involvement of PDCD6 during the miR-9875-mediated apoptosis regulation in mud crab. AMO-miR-9875 were co-injected with PDCD6-siRNA for 48 h, then the hemocytes were subjected to annexin V assay. All the numeral data represented the mean ± s.d. of triplicate assays (**, p < 0.01).

To explore the involvement of miR-9875 during PDCD6-mediated apoptosis regulation, the caspase 3/7 activity and apoptosis rate in mud crabs treated with AMO-miR-9875 and PDCD6-siRNA were determined. The results showed that the caspase 3/7 activity and apoptosis rate were significantly increased in the miR-9875 knockdown mud crabs (Figure 6a and b), indicating that miR-9875 was an anti-apoptotic miRNA. Moreover, in the mud crab co-treated with AMO-miR-9875 and PDCD6-siRNA, the upregulation of apoptosis caused by miR-9875 interference was significantly reduced (Figure 6a and b), demonstrating the involvement of the miR-9875-PDCD6 pathway during apoptosis regulation. Taken together, these data suggested that miR-9875 could suppress apoptosis by targeting PDCD6 in mud crabs.

### *The promotion of WSSV infection by miR-9875* via *targeting PDCD6*

**Figure 7.**
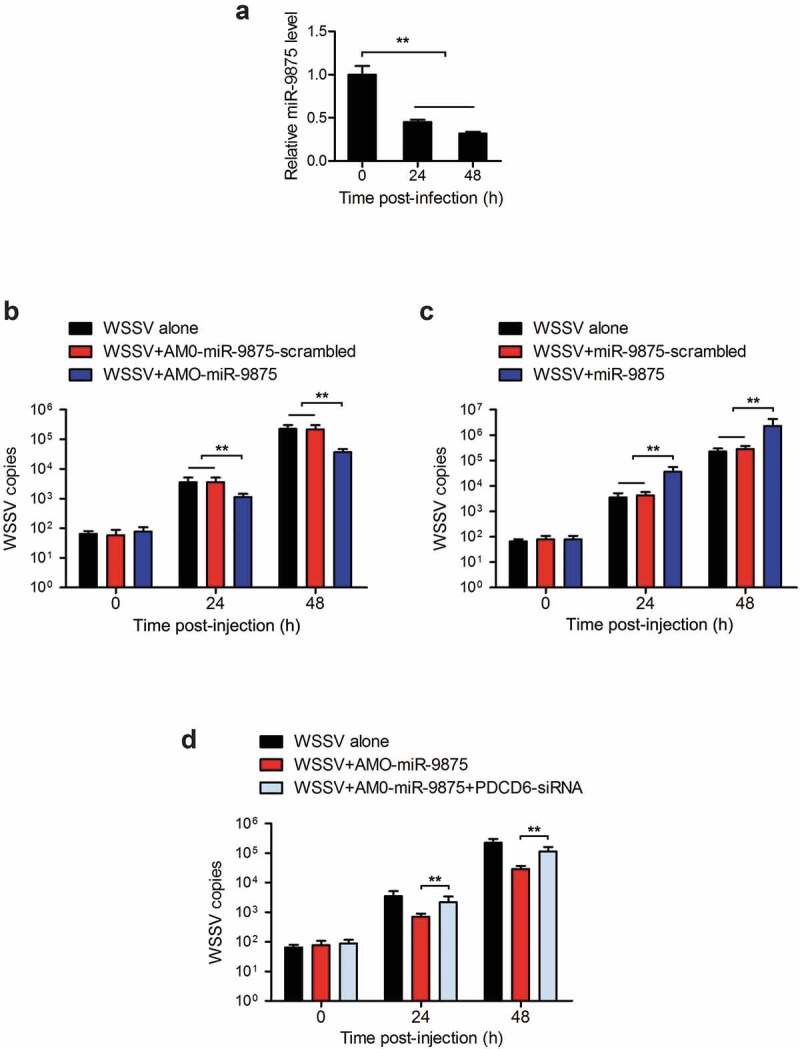
Enhancement of the WSSV proliferation by miR-9875 *via* targeting PDCD6 (a) The detection of miR-9875 expression in mud crabs upon the WSSV challenge using quantitative real-time PCR analysis. (b) Analysis of copy numbers of WSSV in mud crabs treated with either AMO-miR-9875 or AMO-miR-9875-scrambled. (c) The copy numbers of WSSV were detected in mud crabs treated with either miR-9875 or miR-9875-scrambled. (d) The participation of PDCD6 during the miR-9875-mediated virus promotion, AMO-miR-9875, WSSV and PDCD6-siRNA were co-injected into mud crabs, which was followed by the detection of the copy numbers of WSSV. Data presented were representatives of three independent experiments (**, *p* < 0.01).

To determine whether miR-9875 could affect WSSV proliferation in mud crab, mud crab was challenged with WSSV and the expression of miR-9875 was then detected. The expression of miR-9875 was found to be significantly decreased at 24 h and 48 h post-WSSV challenge (Figure 7a), indicating the involvement of miR-9875 in virus infection. To further explore the role of miR-9875 during the virus infection, miR-9875 was overexpressed or silenced in mud crabs, and then the copy numbers of WSSV were evaluated. The results indicated that the copy numbers of WSSV were significantly decreased in miR-9875 silenced mud crabs compared with the controls (Figure 7b). The overexpression of miR-9875 significantly increased the copy numbers of WSSV (Figure 7c), which suggested the positive role of miR-9875 in the virus infection. Furthermore, when mud crab co-treated with AMO-miR-9875 and PDCD6-siRNA, the AMO-miR-9875-mediated virus suppression was remarkably relieved (Figure 7d). Taken together, these data suggested that miR-9875 could promote WSSV infection by targeting PDCD6 in mud crabs.

**Figure 8.**
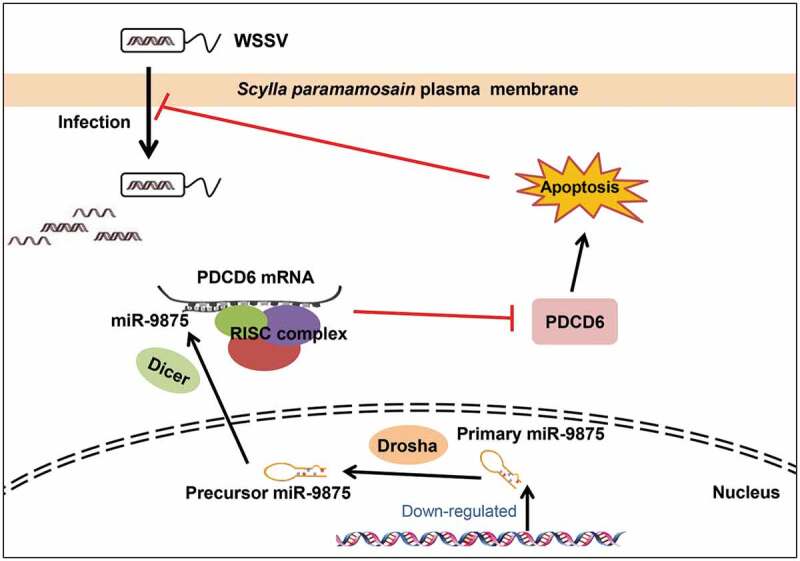
The proposed schematic diagram for the miR-9875-PDCD6 pathway-mediated apoptosis and virus invasion regulation in mud crab.

In summary, the above findings revealed that during WSSV infection, the level of miR-9875 was significantly decreased, resulting in the upregulation of PDCD6 and the occurrence of cell apoptosis in response to virus infection in mud crabs (Figure 8).

## Discussion

It is well known that apoptosis is central to the control and elimination of viral infections [27]. Viruses can lead to the occurrence of apoptosis in the infected cells through triggering cellular sensors that initiate cell death [28]. Respiratory syncytial virus (RSV) could sensitize the infected lung carcinoma cell to apoptosis through the activation of TRAIL [29]. Also, the infection of Type 2 PRRSV (porcine reproductive and respiratory syndrome virus) could induce the apoptosis of B- and T-cells in experimentally infected pigs [30]. In *Litopenaeus vannamei*, during the WSSV infection, the apoptotic ratio of the hemocytes was significantly increased, while this process could be effectively suppressed when ASK1 (apoptosis signal-regulating kinase 1) silenced [31]. At present, the relevant research conducted in marine invertebrates is still limited. In this study, the apoptosis level in mud crabs was found to be remarkably upregulated in response to WSSV infection. Also, the miR-9875-PDCD6 pathway was important in apoptosis and virus infection in mud crabs. This result revealed a novel miRNA-mediated mechanism of the regulation of apoptosis and virus infection.

PDCD6, a member of the penta-EF-hand protein family [32], has been found to be dysregulated in the tumors of various origin and contributed to the viability of cancer cells [33]. As a key apoptotic regulator, PDCD6 is responsible for a p53-responsive gene and the nuclear accumulation of such significantly induced apoptosis in response to DNA damage [34]. Besides, PDCD6 could mediate the pro-apoptotic activity of cisplatin or TNFα through the downregulation of NF-κB expression in human ovarian carcinoma cells [35]. In addition, it has been reported that PDCD6 could interact with DAPk1 (Death-associated protein kinase 1) and regulate apoptosis *via* caspase-3 dependent pathway [9]. So far, PDCD6 is known as a regulator in apoptosis, which is essential in the antiviral immunoregulation; however, the relationship between PDCD6 and virus infection in mud crab has not been previously addressed. In this study, the role of PDCD6 in virus infection was determined, the results showed that the upregulation of PDCD6 was found during the WSSV invasion, and the copy numbers of WSSV was increased in the PDCD6 silenced mud crabs. Our study firstly demonstrated the involvement of PDCD6 in the immune response of mud crabs on virus infection.

RNAi, a natural defensive response to the virus infection, was mainly mediated by siRNAs or miRNAs through post-transcriptional gene regulation [36,37]. It has been reported that miRNAs could negatively regulate the expressions of specific target genes by seed sequence complementary binding to the 3ʹUTR of the target genes [38]. MiRNAs have known to be important regulators in many biological processes [39]. Previous research demonstrated that miRNAs were involved in the regulation of apoptosis in both vertebrates and invertebrates [24,40]. However, the miRNAs that targeting PDCD6 and their biological significances in invertebrates still remain unknown. In the present study, we supplied a regulatory layer at the transcriptional crosstalk between miR-9875 and PDCD6 in mud crab. During the WSSV infection, the level of miR-9875 was significantly decreased, leading to the accumulation of PDCD6, followed by the triggering of apoptosis and then attenuate WSSV replication. In this context, our study presented a clue to explore the involvement of miRNAs during PDCD6-mediated virus suppression.
